# Current Insights Into the Maintenance of Structure and Function of Intervertebral Disc: A Review of the Regulatory Role of Growth and Differentiation Factor-5

**DOI:** 10.3389/fphar.2022.842525

**Published:** 2022-06-08

**Authors:** Bin Lv, Weikang Gan, Zhangrong Cheng, Juntao Wu, Yuhang Chen, Kangchen Zhao, Yukun Zhang

**Affiliations:** Department of Orthopaedics, Union Hospital, Tongji Medical College, Huazhong University of Science and Technology, Wuhan, China

**Keywords:** intervertebral disc degeneration, growth and differentiation factor (GDF), nucleus pulposus, extracellular matrix, mesenchymal stem cell

## Abstract

Intervertebral disc degeneration (IDD), characterized by conversion of genotypic and phenotypic, is a major etiology of low back pain and disability. In general, this process starts with alteration of metabolic homeostasis leading to ongoing inflammatory process, extracellular matrix degradation and fibrosis, diminished tissue hydration, and impaired structural and mechanical functionality. During the past decades, extensive studies have focused on elucidating the molecular mechanisms of degeneration and shed light on the protective roles of various factors that may have the ability to halt and even reverse the IDD. Mutations of GDF-5 are associated with several human and animal diseases that are characterized by skeletal deformity such as short digits and short limbs. Growth and differentiation factor-5 (GDF-5) has been shown to be a promise biological therapy for IDD. Substantial literature has revealed that GDF-5 can decelerate the progression of IDD on the molecular, cellular, and organ level by altering prolonged imbalance between anabolism and catabolism. GDF family members are the central signaling moleculars in homeostasis of IVD and upregulation of their gene promotes the expression of healthy nucleus pulposus (NP) cell marker genes. In addition, GDF signaling is able to induce mesenchymal stem cells (MSCs) to differentiate into NPCs and mobilize resident cell populations as chemotactic signals. This review will discuss the promising critical role of GDF-5 in maintenance of structure and function of IVDs, and its therapeutic role in IDD endogenous repair.

## Introduction

As a major public health concern, low back pain (LBP) is a leading reason for disability worldwide, affecting all-age population. LBP is associated with pathologies including intervertebral disc herniation, spine stenosis, and radiculopathy. Although the etiology of LBP is complex and multifactorial, intervertebral disc degeneration (IDD) has been cited as a leading pathological contributor to LBP. IDD is a common musculoskeletal degeneration that progresses with age, which is the major cause of physical disability in aging populations. Serving as a shock absorber, intervertebral disc (IVD) is the fibrocartilaginous joint positioned between the vertebrae and composed of three structural components, sustaining mechanical strength and conferring spinal flexibility. It is estimated that 84% of the LBP cases experience symptom at least once throughout lives (Ahlholm et al., 2021). Consequently, there has been an increase in efforts to find innovative therapeutic strategies.

**FIGURE 1:**
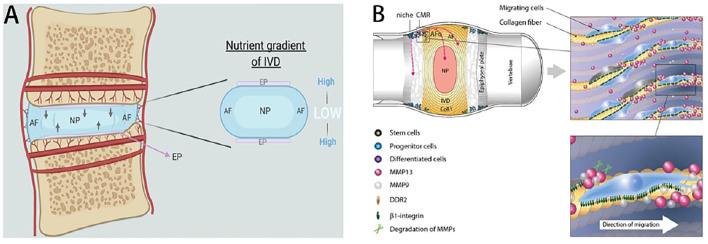
Overview of IVDs structure and the cellular migration/directions during IVDs repair. **(A)** Avascular nature of the human IVDs. Reproduced from Rebecca et al.(Kritschil et al., 2021) with permission from Copyright 2021 Wiley. **(B)** Intervertebral disc stem cell niche and cells migrate to AF, the outer layer of collagen-rich fibers (COLL I). Cells migrate to the germinal region and interact with ECM along the alignment of collagen fibers. Moreover, the disorganization of collagen fibers and increasing of the interlamellar distances between collagen bundles in the annulus fibrosus were usually observed during the degeneration process that often results in disc bulging. Reproduced from Henriksson et al. (2015) with permission from Copyright 2015 Elsevier.

The injection of targeted proteins fully reverses tissue pathology in terms of its structural and functional complexity. The IVD is a complex fibrocartilaginous structure that links adjacent vertebrae and confers spinal mobility. Consisting of the annulus fibrosus (AF), nucleus pulposus (NP), and cartilage endplate (CEP), the IVD is the largest non-vascularized structure where cells retrieve nutrients and oxygen mainly by diffusion through CEP, readily advancing to nutritional deficiency in ageing population. A relatively low number of IVD cells are inactive metabolically, and therefore most of their properties depend on IVD extracellular matrix (ECM). The ECM is physiologically rich in proteoglycans, consisting of a protein core and a multitude of glycosaminoglycan (GAG) side chains, entrapped in the network of collagen fibres. Resident IVD cells are maintained under a high osmotic, hypoxic, and high-load microenvironment. Multifactorial including genetic, molecular, cellular and mechanical overloading contribute to an imbalance between production and degradation of the ECM, initially within the NP and eventually causing IDD (Mayer et al., 2013). Furthermore, increased catabolic processes of glycosaminoglycans (GAGs) decrease the swelling capacity of the NP. Thus, the altered mechanical loading of the IVD leads to vascularization and neoinnervation with infiltration of immune cells. Due to the limited self-repair capacity and harsh nutrient availability of IVD, the ECM degradation process is irreversible and requires restoration if IVD repair is pursued (Figure 1). For reasons that are not yet fully understood, very early after skeletal maturity, the IVD can undergo a degenerative process that manifests as cell death, extracellular matrix (ECM) changes and dehydration, culminating in failure of its biomechanical properties, thereby leading to pain and disability. Thus, they have a limited capacity for self-repair after damage or degeneration (Zhang et al., 2021a). The extracellular matrix (ECM) is mainly synthesized and secreted by NP cells (NPCs) and is composed of type II collagen (collagen II) and aggrecan, helping to resist compression and maintain disc height. Its pathogenesis is very complicated and it involves a variety of pathological processes, including apoptosis, cell proliferation, degradation of the extracellular matrix, inflammation, and degeneration of cartilage endplates, which will lead to complex biochemical and molecular changes in the intervertebral disc, including the reduction of proteoglycan content, the conversion of collagen type II to collagen type I, and the decrease of NP cell density. IDD is considered to arise from cell driven degeneration to the ECM of the central part of the IVD, which eventually result in structural damage, such as AF rupture, NP cells protruding. This process is concurrent with an in-growth of blood vessels and nociceptive nerve fibers into the IVDs, promoting immune cell infiltration and resulting in pain and contributing to the abnormity of IVDs mechanical function (Zhang et al., 2021a).

IDD is mainly characterized by apoptosis of function cells, an imbalance between ECM anabolism and catabolism, and the dysregulation of NPC survival (Wang et al., 2021). IDD leads to imbalance between anabolic and catabolic processes, altered extracellular matrix composition, loss of tissue hydration, aberrant activation of the inflammation, and impaired mechanical functionality. Unfortunately, current treatments are aimed at relieving symptoms instead of preserving disc structure and function. The hallmark of IDD is the gradual loss of ECM molecules-specifically the GAG-substituted proteoglycans. While this loss is often associated with increased extracellular catabolism via metalloproteinases and pro-inflammatory cytokines, evidence suggests that IDD is related to dysregulation of the enzymes involved in GAG biosynthesis.

Any deviation from the homeostatic balance between the anabolic and catabolic factors in the normal, healthy disc will result in degenerative process of IVDs. Because IVD tissue homeostasis is maintained by a balance between anabolic and anti-catabolic process of IVD cells, current strategy to regenerate degenerative IVDs is to promote the anabolism and to suppress the catabolism induced by cytokines. Various growth factors have been demonstrated to shift the catabolic state to the anabolic state to regenerate IVDs. Recently, the injection of growth factors has been under serious consideration as a potential biological therapy to enhance IVD tissue regeneration. The feasibility, safety, and efficacy of injection of growth factors for IVD degeneration has been heavily investigated. We reviewed the role of chosen prototypical growth factors and growth factor combinations that have the capacity to improve IVD restoration. A number of growth factors have showed potential to modulate the anabolism and catabolism through multiple pathogenic mechanisms, including suppressing inflammatory process and down-regulating degrading enzymes, in both *in vitro* and animal studies of IVDs tissue engineering. Previous studies have been generated about the outcomes after the intradiscal administration of a series of growth factors: growth and differentiation factor-5 (GDF-5), bone morphogenetic protein-2 (BMP-2), and bone morphogenetic protein-7 (BMP-7), transforming growth factor beta-1 (TGF-β1), platelet-derived growth factor (PDGF), insulin-like growth factor-1 (IGF-1), basic fibroblast growth factor (bFGF), fibroblast growth factor-18 (FGF-18). Members of the transforming growth factor-β superfamily, IGF-1, GDF-5, BMP-2, BMP-7, and platelet-derived growth factor have all been investigated as possible therapeutic options for IVD regeneration. However, growth factors, including Transforming Growth Factor-β (TGF-β), Fibroblast Growth Factor (FGF), and Insulin-like Growth Factor-1 (IGF-1), may induce blood vessel in-growth and accelerate the process of IDD. Growth factors can be applied in IVD tissue regeneration via delivery of the “naked” or “embedded” proteins as well as prolonged supplement by vector- or cell-based gene therapy (Paesold et al., 2007).

The metabolic activity of IVDs is modulated and regulated by several growth factors, enzymes, and cytokines via either autocrine or paracrine manner (Wang et al., 2021). Studies have showed that injection of GDF-5 into the intervertebral disc of mice can effectively attenuate the IDD, which results in their response via BMPRII and will not stimulate blood vessel ingrowth (Fitzgerald et al., 2021). An increasing body of evidence indicates that GDF family members are central to IVD homeostatic processes and show up-regulation of healthy NP cell marker genes in degenerative cells, induce MSCs to differentiate into NP cells and even act as chemotactic signals mobilizing resident cell populations during disc injury repair. Furthermore, GDF-5 has been determined to induce the restoration of disc height and to increase the proteoglycan (PG) content in NP (Wang et al., 2021). This finding suggests that GDF-5 is more suitable for use in IDD treatment compared with the three other growth factors. In this review, we will focus the discussion on the basic structure, signaling pathways, function in cartilage and bone formation, and potential clinical application of GDF5 in bone tissue regeneration.

### Overview of GDF-5

Growth and differentiation factor-5 (GDF-5), also known as CDMP1 or BMP14, is a divergent member of the transforming growth factor-beta (TGF-b) superfamily and is expressed under physiological conditions. GDF-5 is synthesized as a large precursor protein with seven cysteine residues, which contains two major domains: the N-terminal prodomain with a cleavage site and signal sequence and the active C-terminal domain. GDF-5 binds to two types of transmembrane serine/threonine kinase receptors to activate its signaling pathway (Genovesi et al., 2021). Specificly, upon GDF-5 binding to the extracellular part of the receptor complex, BMP ligands signal through a receptor complex consisting of two type I BMP receptors (BMPRs) and two type II BMPRs to activate the downstream Smad pathway (Xu et al., 2021). This spatialtemporal expression pattern of GDF-5 proves its essential role in the formation of bone and cartilage. Similar to other members of BMPs, the signaling cascade of GDF5 is originated through binding to type I and type II receptors and thus regulating the downstream intracellular biochemical processes. The intracellular Smad proteins become activated and then translocate into the nucleus to regulate the transcription of COL2A1 and ACAN, which encode for type II collagen and aggrecan, respectively (Massagué, 2000). Each BMP ligand has a varying affinity for each receptor subtype and GDF-5 is known to preferentially bind to the BMPR1b type I receptor and BMPR2 type II receptor. Like that of GDF5, these receptors are expressed in the developing rat ventral mesencephalon (VM) at embryonic day 11 and their expression continues throughout development until adulthood, at least up to postnatal day 90. Li et al. (2015) found that GDF-5 and BMPRII expressed both in normal and degenerated IVD, showing that gene therapy may produce be a physiological effects. They showed that GDF-5 might have an inhibition effect on degenerated human IVD.

GDF-5 plays a crucial role in developmental processes of organs including bone, cartilage, ligament, and soft tissue formation. Mutations of GDF-5 are associated with several human and animal diseases that are characterized by skeletal deformity such as short digits and short limbs. GDF-5 is emerging as a major mediator in the interzone of joint formation sites, and in the cartilage primordium in the early limb development. *In vitro* and *in vivo* studies showed that GDF-5 overexpression or treatment of recombinant protein stimulated chondrogenesis and osteogenesis.

Over the past decades, studies regarding the role of GDF-5 in progression of musculoskeletal diseases have been conducted. GDF-5 seems uniquely capable of stimulating beneficial effects on IVD and ECM without inducing ectopic ossification (Belykh et al., 2015).GDF-5-gene-deficient mice demonstrated abnormalities in IVD structure and ECM. Adenoviral mediation of GDF-5 gene can stimulate NPCs growth. For instance, Liang et al. showed the therapeutic effects of gene delivery for intervention for IDD in a mice model by of adenovirus-mediated GDF-5 delivery. Their findings highlighted the physiological improvements that occurred to the IVD and showed the long-term expression of the target protein in the IDD (Liang et al., 2010). GDF-5 was observed in both normal and degenerated human IVD, particularly in the NPCs. In addition, degenerated human IVD cells show reduced number of cells expressing GDF-5. An investigation of polymorphisms in GDF-5 revealed that its variable expression and function are linked to osteoarthritis (Masuya et al., 2007). Williams et al. investigated SNP rs143383 (a T to C substitution at position 104) located within the promoter region of the GDF-5 gene. Their analysis showed that the T allele was associated with 1.72-fold increased risk of disc space narrowing and osteophyte production in women (Zhu et al., 2019). This result shows that GDF-5 is a convincing candidate gene for bone tissue engineering by promoting osteogenesis and angiogenesis.

Jodie et al. (Daniels et al., 2017) suggested that both GDF-5 and IL-1 could activate intracellular ERK signaling pathways in the degenerated IVD. Liu et al. (2016a) showed that microRNA-34a inhibition could increase GDF-5 expression to prevent IL-1β-induced ECM degradation in human NPCs. The commitment of hASCs is powerful and highly specific, as evidenced by the expression of NPCs-associated genes characteristic of normal human NPCs. Pauline et al. (Colombier et al., 2016) showed that the GDF-5 and TGF-β1 synergistically drive the human adipose stromal cells (hASCs) differentiate into NPCs-like cells. Enochson et al. (2014) showed that GDF-5 stimulation of human chondrocytes inhibited expression of the cartilage ECM degrading enzymes MMP-13 and ADAMTS-4 and stimulated the expression of cartilage anabolic genes ACAN and Sex determining region Y-Box9 (SOX9) via the canonical Wnt signaling pathway. Luo et al. (Enochson et al., 2014) showed that GDF-5 gene insertion displayed significant promise for applications in repairing the matrix of degenerated IVD cells (Luo et al., 2016).

### GDF-5 Affects ECM Metabolism in IVD

Physiological NP matrix contains 70–90% water, while its dry weight consists of 20% collagen, mainly type II, and 30–50% proteoglycan. IVDs comprise three regions: a hydrophilic nucleus pulposus (NP), an outer fibrocartilaginous annulus fibrosus (AF), and bordered superiorly and inferiorly by hyaline cartilaginous endplates (CEP). Resident cells in both the NP and AF compartments produce and secrete the complex ECM molecules that accommodate the compressive and tensile mechanical loads, respectively.

Under pathological conditions, the inbalance between the synthesis and decomposition of the ECM decreased structural components (collagen II and aggrecan) and increased matrix-degrading enzymes (MMP-2, MMP-3, MMP-13, ADAMTS-4, and ADAMTS-5). IDD is closely related to the reduction of hydrophilic ECM molecules and the changes in the phenotype of IVD cells, leading to structural changes and instability of the spine (Bedore et al., 2014). ECM degradation is a key factor in the progression of IDD, contributing to loss of compression resistance and IVD height. ECM degradation produces various severe consequences. For instance, decreased levels of aggrecan and collagens cause various characteristic features of IDD, including NP dehydration and fibrosis, AF disorganization, and CEP calcification (Jing and Liu, 2021). In addition, loss of NP proteoglycan matrix coupled with increased breakdown products of proteoglycan attenuates the hygroscopic properties of ECM, leading to decreased water content, swelling pressure and ability to withstand load. Dehydration of the NP also suppresses the availability of nutrients and growth factors to the disc cells, leading to further impairment of IVD function (Bridgen et al., 2017).

Matrix metalloproteinases (MMPs), a family of zinc-dependent endopeptidases, are divided into six categories: collagenases, stromelysins, gelatinases, membrane-type MMPs, and unclassified types. Gelatinases (MMP-2 and MMP-9) degrades denatured collagen, laminin and gelatins. Stromelysins (MMP-3, MMP-10, and MMP-11) break down proteoglycans, collagens and gelatins. Collagen enzymes (MMP-1, MMP-8, MMP-13, and MMP-18) are predominantly cellulosic collagen. Membrane-type MMPs (MMP-14, MMP-17, MMP-24, and MMP-25) are localized to plasma membranes and have cytoplasmic domains. These MMPs play an important role in activation of signal transduction pathway and the other proteases.

MMPs are increasingly recognized as key elements in in disc tissue degradation and re-absorption. The researchers showed that MMPs played a dominant role in IDD and that elevated levels of MMP-2 and MMP-9 were associated with grading of degenerative disc disease. For instance, miR-155 is down-regulated in degenerative NP cells, and MMP-16 increased aggrecan and Col II degradation, resulting in the dehydration and IDD (Zhang et al., 2017). Mouse IVD cells were treated with GDF-5 protein and cDNA, and then IVD cell proliferation, proteoglycan production, and ECM–related gene expression were detected to assess therapeutic effect. GDF-5 can slow down the decomposition of ECM by suppressing the expression of MMP-3 (Tan et al., 2018). Stimulated with rhTGF-beta1, and to a lesser extent with GDF-5, BMSCs proliferated, and synthesized ECM rich in collagen (type I and III) when applied to a 3D hybrid construct (Jenner et al., 2007). Han et al. (2016) showed that aggrecan and collagen (type I and II) were higher expressed after GDF-5 treatment in terms of the mode of GDF-5 in promoting the chondrogenic differentiation of ADSCs. IL-1 treatment dose-dependently reduced GDF-5 gene expression in NP cells. The aggrecan and collagen II synthesis of NP cells were both upregulated after appropriate GDF-5 protein supplement, which is probably based on the mediation of ALK6 (Yang et al., 2020). The injection of GDF5-GMs *in situ* promoted ADSCs differentiation and induced the synthesis of an NP-like coat and ECM, restoring the IDD (Wu et al., 2018). Co-culture of dopamine modified and GDF-5 laden PCL-HA scaffolds and hBMSCs can promote hBMSCs’ adhesion, proliferation, and chondrogenic differentiation (Xu et al., 2018). However, as reflected by increased expression of ECM mRNA levels, fibroblastic differentiation of MSCs is selectively increased in the absence or presence of BMP6 and not GDF-5 under hypoxic conditions (Lui et al., 2021). Hypoxia renders cells more responsive to treatment with BMP6 as reflected by increased expression of ECM mRNA levels. These findings demonstrate that GDF-5 may be a useful growth factor to stimulate proteoglycan production in the human IDD and hence the repair of the ECM (Colombier et al., 2016). While not technically a member of the BMP family, GDF-5 is strictly associated with this family of proteins and has been shown to influence joint and skeletal development via ECM production and IVD cells growth and differentiation (Gruber et al., 2014; Luo et al., 2016).

**FIGURE 2:**
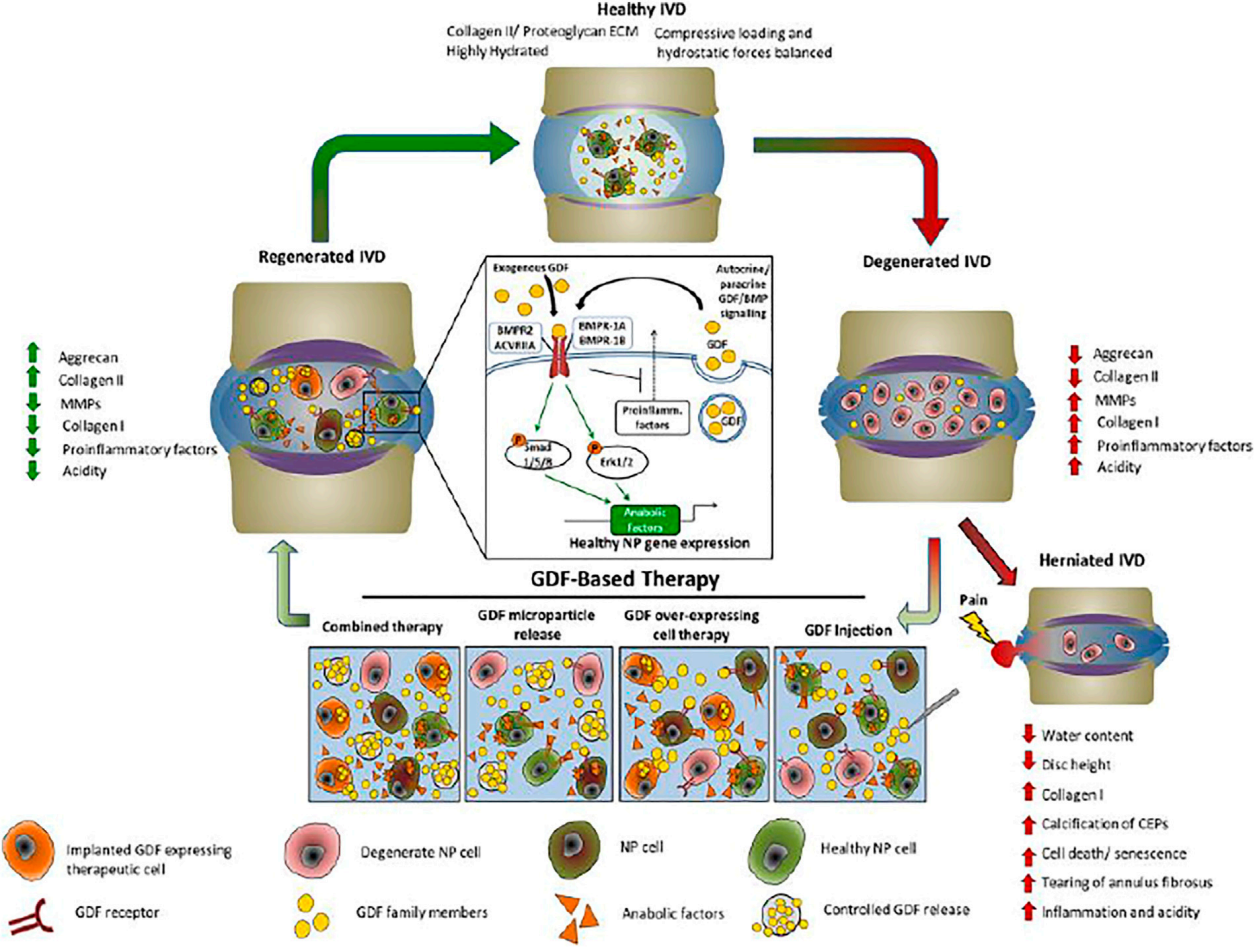
The effect of GDF-based therapeutic cycle in IDD. The imbalance between synthesis and catabolism in normal IVDs and degenerate IVDs increase inflammation and alter ECM production. The processes accelerate increasing type I collagen and decreasing proteoglycan production, compromises tissue integrity, and cause NP herniation and CLBP. Moreover, injection of GDF has shown promise for readdressing the balance between catabolic and anabolic processes in the degenerate IVD, thereby restoring disc matrix integrity and function, and reducing pain. ECM, extracellular matrix; GDF, growth differentiation factor; IVD, intervertebral disc; MMP, matrix metalloproteinase; NP, nucleus pulposus. Reproduced with permission (Hodgkinson et al., 2019). Copyright 2019, Wiley.

GDF-5 has been regarded as the main regulator of ECM composition due to their capacity in enhancing production of beneficial components of the ECM while decreasing matrix metalloproteinase expression (Feng et al., 2015). Endogenously, GDF-5 expression is constant throughout different stages of degeneration (Li et al., 2015). GDF-5 is able to exert beneficial effects on IVDs and ECM without inducing heterotopic ossification because GDF-5 shows osteo-inductive activity only at high concentrations. Leslie Frapin et al. (2020) found that pullulan microbeads (PMBs) loaded with CCL5/TGF-β1/GDF-5 constitute an innovative and promising strategy for promoting cell recruitment and ECM. After injection of GDF-5 plasmid nano-microspheres were conducted in rabbits, the ECM proteins in chondrocytes was significantly increased (Chen et al., 2018). Liu et al. showed that miR-132 stimulated ECM degradation around human NP cells by direct targeting of GDF-5 and represented a therapeutic target for IDD treatments (Liu et al., 2017). Liu et al. demonstrated that miR-7 contributes to an impaired ECM in IVD through targeting GDF-5 and prevent IDD (Liu et al., 2016b) (Figure 2).

### GDF-5 Promotes the Proliferation of IVD Cells

Many studies have assumed that a decreased cell count and ECM degradation are highly associated with the behavioural alterations of IVD cells. Induced by various growth factors including TGFβ, IGF-1, FGF-2, and PDGF, MSCs have been modified towards NP-like differentiation in an effort to promote their therapeutic utility. However, current studies pay attention to general chondrogenic markers rather than specific NP differentiation markers. GDF-5 has been investigated as inducers of the expression of general chondrogenic genes (type II collagen; SOX9; ACAN) and, importantly, to also induce specific NP differentiation genes (SHH, KRT18, KRT19, CA12, CD24, HIF1α, and Glut-1) in MSCs. Although a significant increase in markers of hypertrophy and ossification including ALP, collagen types I and X, and OPN was observed, co-culture of high-density of MSCs together with a GDF-5 is therefore required for increased chondrogenic differentiation. However, sparse evidence showed a similar response to GDF-5 in NP cells, indicating an undesirable chondrogenic hypertrophy toward endochondral ossification. The injection of GDF-5 loaded microspheres leads to a restoration of disc height, improvement of sulfated glycosaminoglycan, DNA content, and increased mRNA levels of collagen type II (Yan et al., 2014). GDF-6 stimulates greater expression of NP-marker genes and PG production than GDF-5 in both MSCs and ASCs (Gantenbein-Ritter et al., 2011). Delivery of GDF-5 and GDF-6 through intradiscal injection into IDD models has shown promising results. For instance, in murine models, IDD after static compression showed signs of improvement with a single GDF-5 injection (Masuda et al., 2004). The number of cells expressing both aggrecan and collagen II increases significantly in the NP and AF. Similarly, delivery of GDF-5 improved disc height and histological appearance in stab models of IDD in rabbit (Chujo et al., 2006). The presence of GDF-5 was observed to colocalize with proliferating cells adjacent to the epiphyseal plate (Henriksson et al., 2012). GDF-5 suppress the expression of MMP-13 and ADAMTS-4 in human chondrocytes and expression of pro-inflammatory markers including TNF-α, IL-1β, and prostaglandinE2 (PGE2) in murine NP cells (Enochson et al., 2014). Increased miR-665 expression decreased expression of aggrecan and Col II and promoted NP cell proliferation. Furthermore, ectopic expression of miR-665 increased expression of MMP-3 and MMP-13 through suppressing GDF-5 expression in NP cells (Tan et al., 2018).

### GDF-5 Inhibits Inflammation

Elevated inflammatory factors (TNF-α and IL-1β) levels have been showed to stimulate and deteriorate the development of IDD. Inflammatory molecules and signaling pathways are regarded as major contributors to the onset and development of IDD. Furthermore, the process of IDD is accompanied by an increasing of inflammatory mediators (TNF, IL-1α, IL-1β, IL-6, and IL-17) by IVD cells, which has been implicated in disc herniation, nerve irritation, and in-growth. Neurogenic factors produced by the IVD promote the expression of pain-related cation channels of the dorsal root ganglion, and the depolarization of these ion channels may induce discogenic and root-induced pain. These cytokines trigger a series of pathogenic responses by the IVD cells that can stimulate autophagy and apoptosis. They can not only up-regulate a variety of catabolic mediators that include ADAMTS-4/5, MMP-1, -2, -3, -13, -14, and can disrupt the balance of IVD tissue anabolism and catabolism, promote the degradation of extracellular matrix, which induce structural changes and spinal instability. Chemokines can promote the infiltration and activation of immune cells, thereby expanding the inflammatory cascade. The nuclear factor-κB (NF-κB) signaling pathway is implicated in various complex biological processes including inflammatory responses. The canonical pathway and non-canonical pathway contributing to the activation of the NF-κB signaling cascades. The inner region of the IVD is composed of collagen II and proteoglycans, inhibiting the NF-κB signaling pathway regulated the catabolism of IDD collagen II and aggrecan (Zhang et al., 2021b). Inflammatory mediators had degraded collagen and proteoglycan, although they had been substantially repaired. So inflammatory mediators exhibited a strictly association with IDD, with the mechanism complicated, which results in the promotion of process of IDD in multiple ways. Some of the key pro-inflammatory cytokines, such as TNF-α, IL-1α, IL-1β, IL-6, and IL-8, are found and released at sites of tissue injury which is mediated by NF-κB. Several studies have showed that GDF-5 inhibits the NF-κB signaling pathway, which reduces inflammatory factor gene transcription and diminishes inflammatory factor expression. IL-1 is naturally found within the IVD and is responsible for indirectly degrading ECM components through the production of degradative enzymes, upregulation of other cytokines, and preventing the production of ECM components. A delicate homeostasis is maintained by pro-inflammatory and anti-inflammatory subtypes of IL-1 *in vivo*, which is gradually disturbed by IDD and genetic polymorphisms.

**FIGURE 3:**
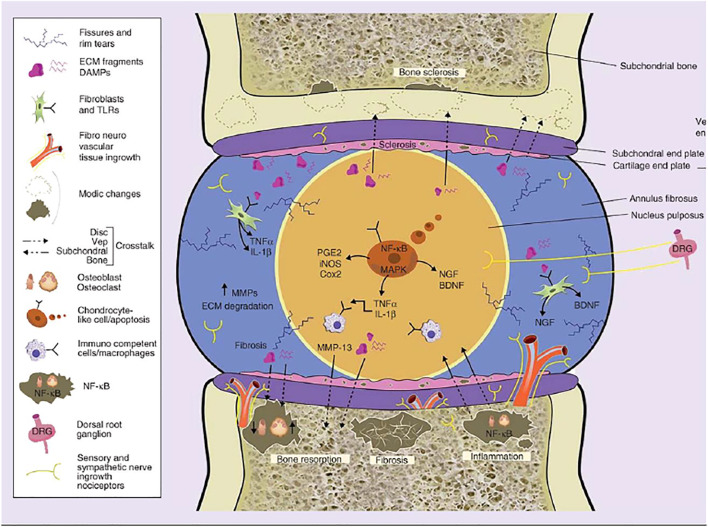
The molecular mechanisms of low-grade inflammation in IVDJD and the crosstalk between intervertebral disc and subchondral bone. Reproduced from Eduardo et al. (Anitua and Padilla, 2018) with permission from Copyright 2018 Future Medicine.

Overexpression of GDF-5 in NP cells also dramatically decreased the protein expression levels of TNF-α, IL-1, PGE2, iNOS, COX-2, collagen-II, aggrecan, IκBα, and p-p65. Accordingly, GDF-5 suppressed the production and release of inflammatory components, relieving LPS-induced IDD. Meanwhile, IL-1β and TNF-α were both found to decrease GDF-5 expression significantly in AF cells in 3D culture (Gruber et al., 2014). The nanoparticles loaded with the dexamethasone and GDF-5 effectively inhibited proliferation of activated macrophages, indicating apoptotic induction and an anti-inflammatory effect (Wu et al., 2021) (Figure 3; Table 1).

**TABLE 1:** Effect of GDF5 on MSCs in culture.

GDF5 Concentration	Cell population	Culture duration	Culture conditions	Outcomes	References
100 μg/ml	hASCs	28 Days	Pullulan microbeads	Promote cell recruitment and ECM remodelling	Frapin et al. (2020)
100 ng/ml	hMSCs	18 Days	3D alginate bead	i) Up-regulate aggrecan and collagen II.	Gantenbein-Ritter et al. (2011)
				ii) Promote expression of aggrecan in relation to collagen	
100 ng/ml	hACs and hMSCs	21 days	GE-silk fleece	GE-silk scaffold thrive hMSC towards a NP-cell-like phenotype or maintain the phenotype of native hAFC.	Frauchiger et al. (2018)
100 ng/ml	ADSCs	14 days	PNIPAAM-g-CS	i) Hydrogel support NP regeneration	Christiani et al. (2021)
				ii) PNIPAAM-g-CS supports the survival, proliferation, and differentiation of ADSCs toward an NP-cell-like phenotype	
100 ng/ml	hACs and hMSCs	4 weeks	2D	i) Up-regulate chondrogenic gene expression in both hACs and hMSCs	Murphy et al. (2015)
				ii) Induce the upregulation of chondrogenic genes and synthesis of cartilage-specific matrix	
				iii) Yield mechanically robust cartilage rich in collagen II and GAGs	
50–500 ng/ml	hMSCs	21 Days	Pellet	i) Upregulate collagen II.	Coleman et al. (2013)
				ii) Potential synergistic relationship with TGFβ1 in driving chondrogenic differentiation	
10 ng/ml; 100 ng/ml; 1,000 ng/ml	AD-MSCs	14 Days	type I collagen hydrogels	i) Differentiate to an NP-like phenotype and results in a more proteoglycan-rich matrix	Clarke et al. (2014)
				ii) GDF6-treated AD-MSCs have a less-stiff matrix composition	
				iii) Induce a matrix that is more akin to the native NP-like tissue	
100 ng/ml	hMSCs	Up to 7 Days	HA-pNIPAM	Support hMSC differente toward the disc phenotype	Peroglio et al. (2013)

### GDFs Promotes the Differentiation of MSCs Toward an NP-cell-like Phenotype

Different types of both GDF and PDGF (in combination or not) have been shown to promote ADSC tenogenesis and cellular proliferation (Luo et al., 2009). *In vivo* and *in vitro* results illustrate that the growth factor injections has the potential to restore IDD at an early stage (Walsh et al., 2004). GDF-5 is involved in the development, maintenance and repair of cartilage and other musculoskeletal soft tissues. Similarly, the NP tissue demonstrates similar histological and biological characteristics. Moreover, a gene mutation in GDF-5 is related to IDD, as previously delineated (Williams et al., 2011). Combination of MSCs and GDF-5-augmented fibrin hydrogel was used to glue collagen and hydrogel constructs onto bone disks to stimulate ALP activity *in vivo* model (Diederichs et al., 2018). Henry et al. (2017) propose injectable Si-HPMC/Pullulan microbeads hydrogel system for the GDF-5 and TGF-β1 delivery in IDD regenerative medicine. Du et al. showed that SOX-9 and GDF-5 co-transfected MSCs differentiated into KRT19-positive NP cells (Du et al., 2015). Notably, a consistent and potentially exploitable response during chondrogenesis of mesenchymal stem cells from osteoarthritis patients to the protein encoded by the susceptibility gene GDF-5 (Ratnayake et al., 2017). MSCs differentiated to an NP-like phenotype following direct co-culture with both nondegenerate and degenerate NP, as shown by upregulation of GDF-5, TGF-β1, IGF-1 and CTGF. Direct co-culture of MSCs with degenerated NP cells lead to upregulate matrix gene expression, accompanied by upregulation of TGF-β and GDF-5 gene expression (Strassburg et al., 2010). Yin et al. (2017) showed that miR-615-3p could inhibite the osteogenic differentiation of lumbar ligamentum flavum cells through suppression of osteogenic regulators GDF-5 and FOXO1. Henriksson et al. (2012) showed that a cellular MR from the IVD stem cell niche resulted in regeneration of the adult mammal IDD. They found that the presence of GDF-5, SLUG, SNAI1, SOX9, and β1-INTEGRIN was observed in the outer AF among rabbits in all age groups, indicating a gradual migration of cells. According to Christian et al. (Bucher et al., 2013), the GDF-5 gene transfer resulted in increased aggrecan and SOX9 expression, as well as more proteoglycans expressed in the GAG/DNA ratio. In addition, the GAG/DNA ratio was somewhat recovered in GDF-5 transfected MCS put into an IDD degeneration model. GDF-5 stimulates Smad 1/5/8 signaling, which improves chondrocyte phenotype. Furthermore, GDF-5 boosted aggrecan gene expression in chondrocyte pellet cultures while having little effect on collagen type X expression (Ayerst et al., 2017). Knocking down of GAS5 suppressed the osteogenic differentiation of hPDLSCs, whereas overexpressing GAS5 bolstered GDF-5 expression and boosted the phosphorylation of JNK and p38 in hPDLSCs (Zhang et al., 2021b). ADSCs could differentiate to the NP cell phenotype with a upregulation of multiple genes and proteins in pertinent growth factors (GDF-5, TGF-b1, IGF-1, and CTGF), and relative NP markers (HBB, FOXF1 PAX1 and CA12), ECM (ACAN, COL2A1, COL6A2 and SOX9). In addition, the expression of gene (COL2A1, ACAN, and COL6A2) in degenerate NP cells was also up-regulated (Wu et al., 2021). MSCs and chondrocytes from the central region of the IVD promote production of proteoglycans, collagens and other matrix proteins, making them promising candidates as cell-based IVD repair. MSCs transfected with the GDF-5 gene were added to an IVD organ culture and generated an insignificant amount of glycosaminoglycan (Bridgen et al., 2017).

**FIGURE 4:**
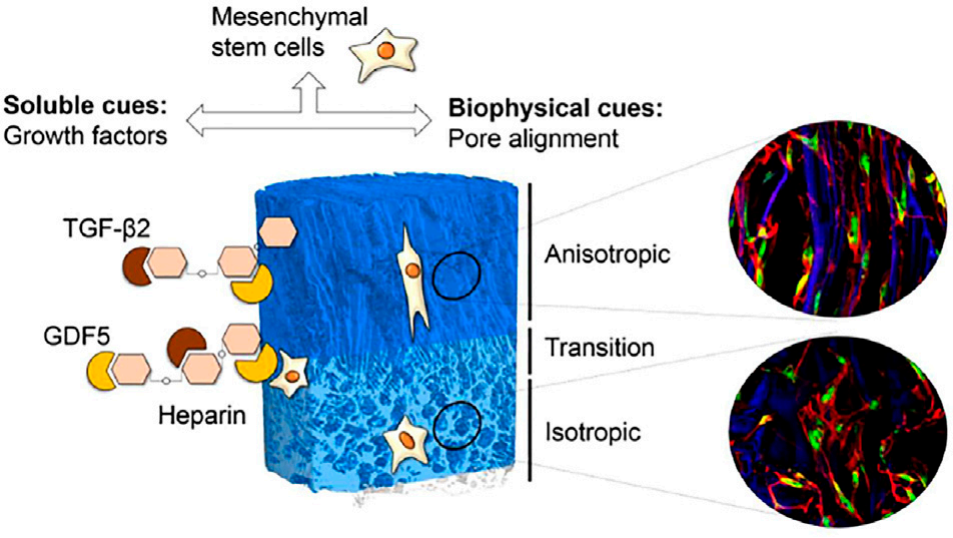
Delivery of GDF-5 with functionalized scaffolds and their role in IVDs repair. The combined impact of GDF-5 and pore alignment can drive MSCs differentiate towards the desired NP cells phenotypes for IDD repair. Engineered scaffolds guide spatial positioning of GDF-5 at specific regions and the temporal control of quantal growth factors release. The delivery of GDF-5 enhanced the expression of markers and collagen II protein content on substrates with isotropic porosity. Reproduced from Font Tellado et al. (2018) with permission from Copyright 2018 Elsevier.

MSCs are capable of self-renewal and multipotential characteristics, allowing them to differentiate into certain mesenchymal and non-mesenchymal lineages (Le Maitre et al., 2009). Beside, the differentiation of MSCs depends on various biological factors. The differentiation of MSCs into NP-like cells has been induced by the growth factors including TGF-β, IGF-1, FGF-2, and PDGF (Xia et al., 2019). These factors can activate proliferation and differentiation of endogenous stem or progenitor cells, stimulate the expression of ECM proteins, regulate the pro-inflammatory cytokines expression and suppress apoptosis and scarring of the remaining tissue (Novoseletskaya et al., 2020). MSCs are also considered to secrete a wide range of bioactive factors including ECM components to modulate the microenvironment at the primary site of action, referred to as “trophic activity” (Miranda et al., 2019). MSCs transplantation can increase the cells number and the ECM accumulation (Lu et al., 2008). NP cells can release soluble factors to direct differentiation of ASCs, which combinated with a nucleus-mimicking collagen II microenvironment enhances differentiation towards NP cell lineage. Influence of collagen type II and nucleus pulposus cells on aggregation and differentiation of adipose tissue-derived stem cells. For instance, GDF-5 can induce ADSCs differentiate into an NP-like phenotype (Zhu et al., 2019). However, the carrier might determine the fate of MSCs without the presence of GDF-5. For instance, MSCs cultured in a HA-pNIPAM under hypoxia can differentiate toward an NP-like phenotype, regardless of whether GDF-5 was added (Peroglio et al., 2013). Moreover, MSCs suspended in HA-pNIPAM without GDF-5 demonstrated stronger NP-like differentiation than MSCs pre-differentiated with GDF-5 in HA-pNIPAM in a bovine caudal disc organ culture model (Peroglio et al., 2013). This increased ratio in PG composition of ECM in comparison to collagenous matrix is a central property of NP tissue and is required for correct functionality. Importantly, reports to date indicate no increase in collagen X production in GDF-6 stimulated cultures as seen with other chondrogenic factors. This lack of hypertrophy and progression toward endochondral ossification when using GDF-6, coupled with the enhanced expression of NP markers, PG production and the higher aggrecan to type II collagen ratio observed in comparison to GDF-5 strongly suggests that GDF-6 is the most promising candidate to produce implantable NP cell phenotypes from MSCs or particularly ASCs. Xia et al. (2019) showed that the combination of GDF-5-loaded gelatin microspheres and NP-like cell could promote regeneration of the IVD after transplantation into rat coccygeal intervertebral discs. PMBs loaded with GDF-5/CCL5/TGF-β1 constitute an innovative sequential release system to promote hASCs recruitment and ECM remodeling (Frapin et al., 2020). This finding is confirmed using the self-assembling method to generate robust, scaffold-free neocartilage constructs using expanded hACs and MSCs. GDF-5, TGF-β1, and BMP-2 stimulation induces chondrogenesis in expanded human articular chondrocytes and marrow-derived stromal cells (Murphy et al., 2015). Human umbilical cord-mesenchymal stem cells (hUC-MSCs)-derived chondroprogenitors demonstrated expression of GDF-5, indicating better regeneration potential as compared to normal MSCs in an IDD model (Ekram et al., 2021). Benjamin et al. (Gantenbein-Ritter et al., 2011) found that GDF6 promotes greater expression of NP-marker genes and stimulates greater PG production than GDF-5 in MSCs. This increased ratio in PG composition of ECM in comparison to collagenous matrix is a central property of NP tissue (Clarke et al., 2014). Liang et al. (2010) injected an adenoviral vector carrying the GDF-5 gene into IVD and found that the gene was successfully expressed and active GDF-5 produced, which result in significant restoration of histology, and improved disc hydration as assessed through MRI. Consistently, adenoviral mediated GDF-5 delivery not only improves the expression of ECM proteins but also could promote the construction and function of NP cells at the level of transcription, translation and post-translation of ECM proteins (Feng et al., 2008) (Figure 4).

### Limitations and Future Prospects

The aim of this review is to provide a review on the regulatory role of GDF-5 in IVD. Gene therapy for degenerative disc diseases is a promising area of research. Insertion of the GDF-5 gene demonstrates promise for applications in repairing the matrix of degenerated IDD. The aim is that the injection of growth factors could surmount the complex degenerative IVD phenotype. The inflammatory environment of the IDD is related with the convergence of GDF and cytokine signaling on kinase cascades. Moreover, the decreased presence of resident cells in IDD tissues renders GDF-5 a candidate for treating painful IDD where a pool of progenitor cells is still retained in the NP and AF. The possible limitations of GDF-5 could be speculated as follows. The mechanism of action of GDF-5 in IDD is very complex, and the study of many signaling pathways is still unclear. GDF-5 may not ultimately work in the avascular intervertebral disc tissue, and the effective dose of injection may be much higher than that of normal physiological agents due to the avascular intervertebral disc tissue. GDF-5 participates in multiple cellular processes in a time-dependent and stage-dependent manner, GDF-5 could be widely used as a therapeutic agent in the musculoskeletal system, indicating that it may help approaches for more functions in IDD. All these limitations suggest that single GDF-5 treatment might not reach its full effects. However, there are few literature reports on whether it can promote the proliferation of BMSCs. GDF-5 induce NP-like differentiation of MSCs, which is promising graft cells for IDD. The combination ofGDF-5 and MSCs and the manipulation of the tissue microenvironment allowing IDD to better respond to GDF-5 offer novel insights into the application. Moreover, to guarantee that the therapeutic effects of GDF-5 are sustained, any approach to improve the safety and feasibility of GDF-5 should be investigated.
